# Complete mitochondrial genome sequence of the white root rot pathogen *Dematophora necatrix* (Xylariaceae: Xylariales)

**DOI:** 10.1080/23802359.2024.2403411

**Published:** 2024-09-12

**Authors:** Magriet A. van der Nest, Emma T. Steenkamp, Lieschen De Vos, Raven Wienk, Velushka Swart, Noëlani van den Berg

**Affiliations:** a Hans Merensky Chair in Avocado Research, Forestry and Agricultural Biotechnology Institute (FABI), University of Pretoria, Pretoria, South Africa; b Department of Biochemistry, Genetics and Microbiology, Forestry and Agricultural Biotechnology Institute (FABI), University of Pretoria, Pretoria, South Africa

**Keywords:** tRNA gene clusters, homing endonucleases, N-acetyltransferase, Xylariaceae

## Abstract

The mitochondrial genome of *Dematophora necatrix* is 121,350 base pairs in length with a G + C content of 30.19%. Phylogenetic analysis showed that *D. necatrix* grouped with other members of the Xylariaceae, with which its mitogenome also shares a broadly similar architecture and gene content. The *D. necatrix* mitogenome contains 14 protein-coding and 26 tRNA-encoding genes, as well as one copy each of the *rnl*, *rns*, *rps3* and *nat1* genes. However, as much as 80% of this genome is intronic or non-coding. This is likely due to expansions and rearrangements caused by the large number of group I introns and the homing endonucleases and reverse-transcriptases they encode. Our study thus provides a valuable foundation from which to explore the mitochondrion’s role in the biology of *D. necatrix*, and also serves as a resource for investigating the pathogen’s population biology and general ecology.

## Introduction

**Figure 1.**
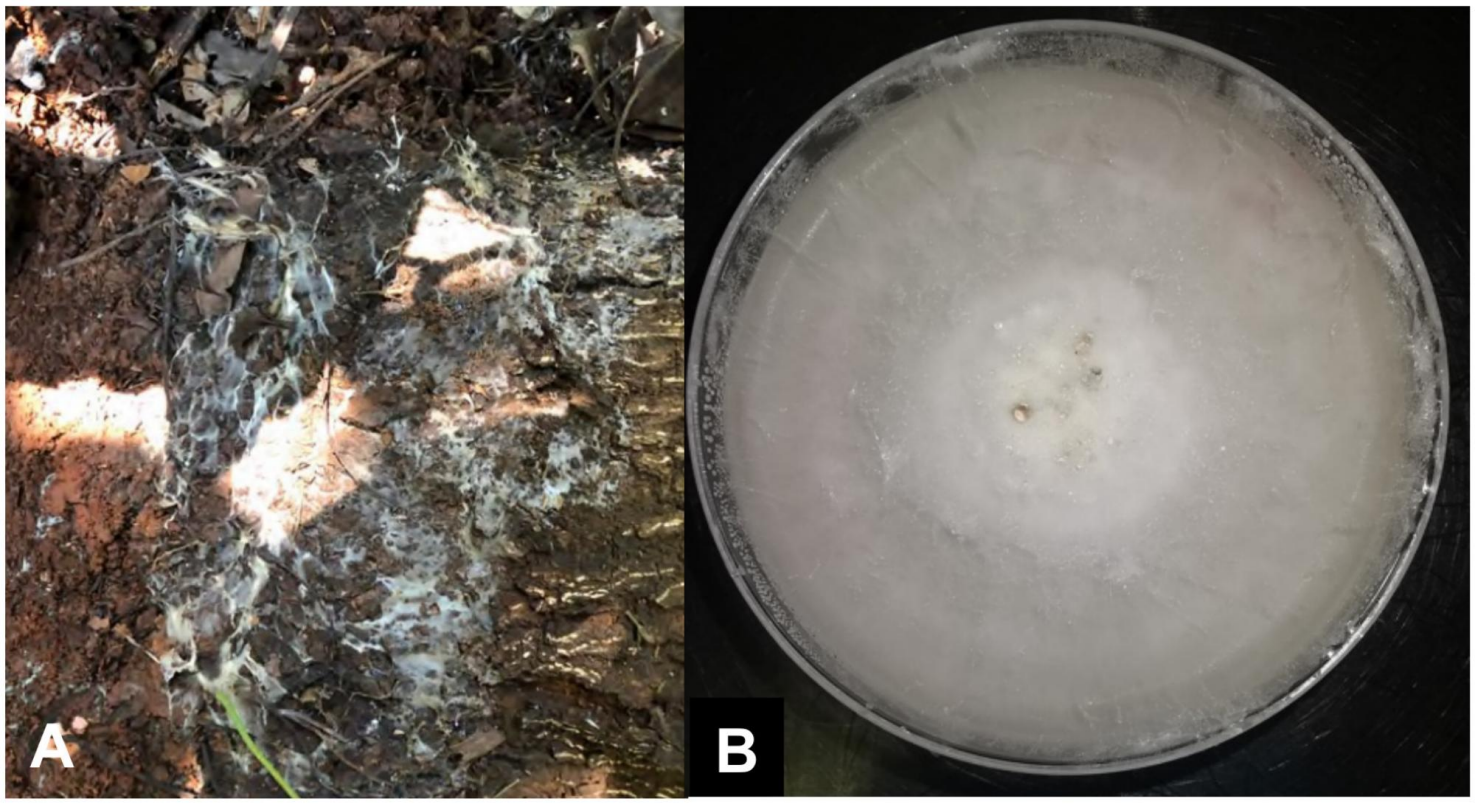
Morphological observation of *dematophora necatrix*. Avocado tree roots colonized with *D. necatrix*, forming a white mycelial mass characteristic of the white root rot disease **(a)**. Mycelial growth cultured on PDA medium at 25 °C for 2 weeks **(B).** Photos taken by raven wienk.

*Dematophora necatrix* Berl. ex Prill. 1904 (Ascomycota, Sordariomycetes, Xylariales, Xylariaceae), also known as *Rosellinia necatrix*, causes the destructive white root rot (WRR) disease of various plant species (Sawant et al. 2021). In avocado (*Persea americana* Mill.), *D. necatrix* hampers production due to susceptibility of rootstocks to WRR (Figure 1A and 1B) (López et al. 2008; van den Berg et al. 2018; Martínez-Ferri et al. 2019). Severity of the disease is further compounded by the pathogen’s resistance to drought and various fungicides (Pérez-Jiménez 2006; Pliego et al. 2009; Magagula et al. 2021). Consequently, *D. necatrix* remains a major concern in avocado-growing regions, globally (van den Berg et al. 2018; Zumaquero et al. 2019).

Effective strategies for curbing the pathogen’s establishment and spread require detailed knowledge regarding its pathogenesis mechanisms, population biology and general ecology. As a result, whole genome sequences for several *D. necatrix* strains have been published (Shimizu et al. 2018; Chavarro-Carrero et al. 2024, including one obtained from a diseased avocado tree in South Africa (Wingfield et al. 2022). Despite the availability of these resources, an annotated assembly for the mitogenome of this fungus is not available. Therefore, the aim of the current study was to assemble and annotate the mitogenome for the South African strain of *D. necatrix*.

## Materials and methods

Strain CMW50482 of *D. necatrix* was collected from a symptomatic avocado tree in the Limpopo province (GPS coordinates: 23°44'59.5"S 30°08'02.4"E) of South Africa (Wingfield et al. 2022). A specimen (voucher number CMW50482) was deposited in the culture collection of the Forestry and Agricultural Biotechnology Institute (University of Pretoria) (https://www.fabinet.up.ac.za/index.php/research-groups/fungal-culture-collections) curated by Dr Seonju Marincowitz (Seonju.Marincowitz@up.ac.za).

Whole genome shotgun sequences (251 bp paired-end reads) for strain CMW50482, which we previously generated using Illumina HiSeq (Wingfield et al. 2022), were used in this study. The mitogenome was assembled using NOVOPlasty v4.3.1 with default parameters (Dierckxsens et al. 2017). The *de novo* assembly was annotated using mitochondrial genetic code 4 and GeSeq - Annotation of Organellar Genomes tool (Tillich et al. 2017) with the following parameters: circular sequence, mitochondrial sequence source, 25% BLAST protein search identity and 85% identity for BLAST rRNA, tRNA and DNA search, third party tRNA annotator ARAGORN v1.2.38 and tRNAScan-SE v2.0, and *Annulohypoxylon stygium* (NC_023117) as Refseq choice. We then used MFannot v1.0 (Lang et al. 2023) and mitochondrial genetic code 4 to assess the gene predictions, while open reading frames (ORFs) and introns were verified using BLAST analyses (https://blast.ncbi.nlm.nih.gov) and the ExPASy translation tool (http://web.expasy.org/translate/).

Size and coding content of the *D. necatrix* mitogenome were compared to those assembled for other Xylariales species using data from GenBank (https://www.ncbi.nlm.nih.gov). Also, protein-coding genes typically found in fungal mitogenomes (Sandor et al. 2018) were subjected to maximum-likelihood (ML) phylogenetic analysis. Here, the inferred protein sequences for *atp6,8,9*, *cox1,2,3*, *nad1*,*2*,*3*,*4*,*4L*,*5*,*6* and *cob* were used. Following alignment with the stand-alone version of MAFFT (–thread 10 –auto –reorder –adjustdirection), the sequences were concatenated using FASconCAT-G v1.04 (Kück and Longo 2014). The concatenated dataset consisted of our *D. necatrix* sequences, as well as those for 25 other filamentous Ascomycota for which relevant data were available in GenBank. Maximum Likelihood (ML) phylogenetic analysis was conducted with IQ-TREE 2 v2.2.2.6 (Minh et al. 2020) using the LG model (Le and Gascuel 2008), while MEGA v11.0 (Tamura et al. 2021) was used for Neighbor-Joining (NJ) phylogenetic analysis based on Poisson distances with rate uniformity among sites. In both cases, branch support was estimated using 1,000 bootstrap replicates.

## Results

**Figure 2.**
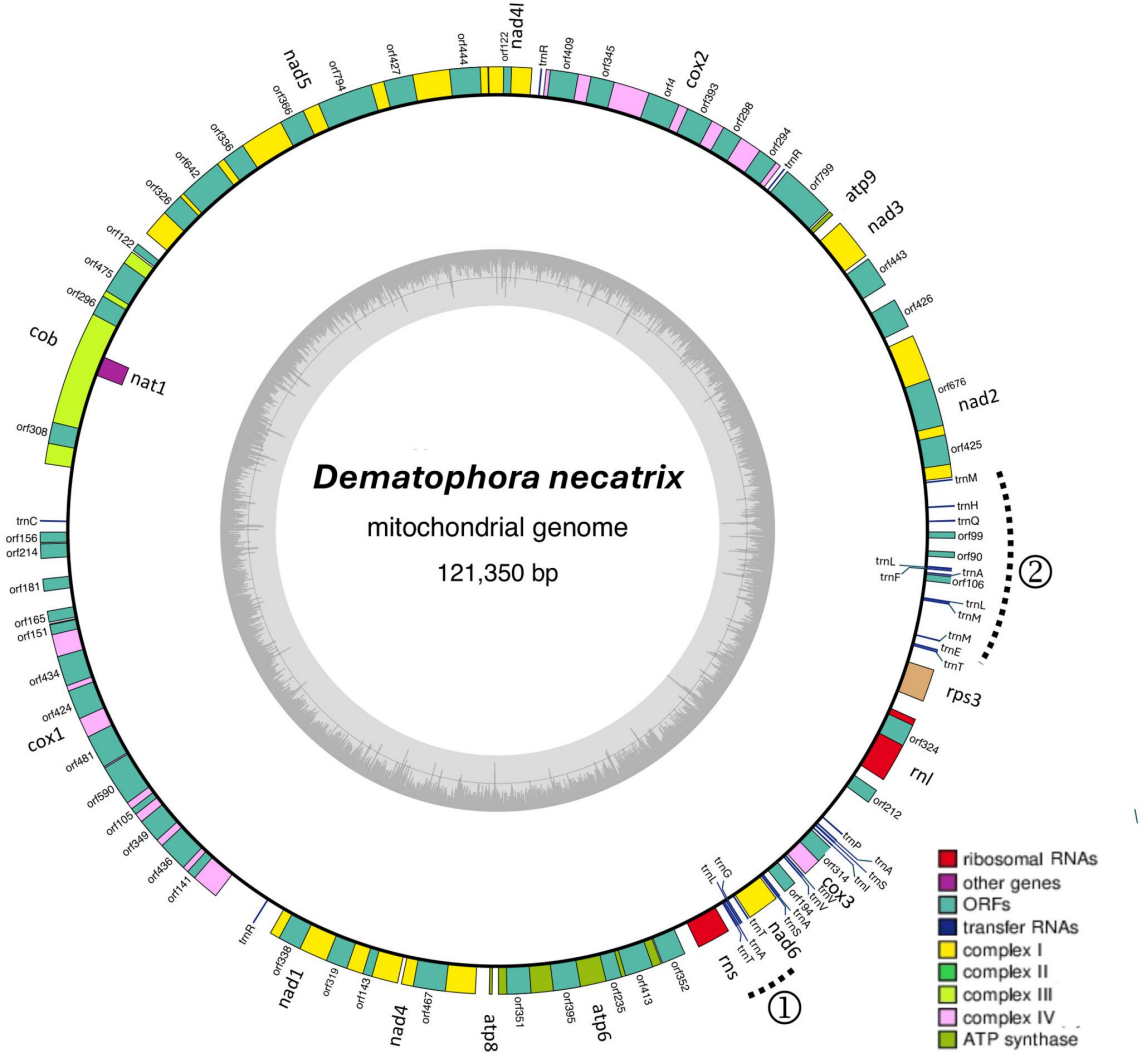
Circular map of the mitochondrial genome of *dematophora necatrix* prepared using OGDRAW program (https://chlorobox.mpimp-golm.mpg.de/OGDraw.html). Genes are color-coded by their functional classification. Genes on the outside of each ring indicate that they are on the forward strand, while genes within the ring indicates those located on the reverse strand. The inner, grayscale bar graph shows G + C content (%), with the Middle line marking the 50% threshold. The positions of the two main clusters of tRNA genes are indicated with the dotted brackets (see supplementary figure S2 for details).

The *D. necatrix* mitogenome assembled as a circular DNA molecule consisting of 121,350 bp (Figure 2). The G + C content averaged at 30.19%, with mean base compositions for A, C, G, and T of 35.4%, 13.3%, 16.9%, and 34.4%, respectively. The average coverage depth was 3622x (Figure S1).

The *D. necatrix* mitogenome contained the 14 expected protein-coding genes. These included genes encoding the cytochrome oxidase subunits of Complex IV, apocytochrome b of Complex III, NADH dehydrogenase subunits of Complex I and the ATP synthase subunits (Figures 2, S2A and S2B). The assembly also contained genes encoding ribosomal protein S3 (*rps3*) and N-acetyltransferase (*nat1*). In terms of RNA coding genes, the mitogenome contained the large and small subunit ribosomal RNA (rRNA) genes *rnl* and *rns*, respectively, as well as 26 transfer RNA (tRNA) genes that mostly clustered at two regions (Figures 2, S2B and S2C). The tRNA genes occurred as single copies, except for the tRNA-Arg (four copies) and tRNA-Val (two copies) and tRNA-Met genes (three copies) (Figures 2, S2D). A total of 22 introns were detected, of which two represented group II introns. The rest were group I introns and contained ORFs coding for homing endonucleases or reverse-transcriptases (Table S1).

**Figure 3.**
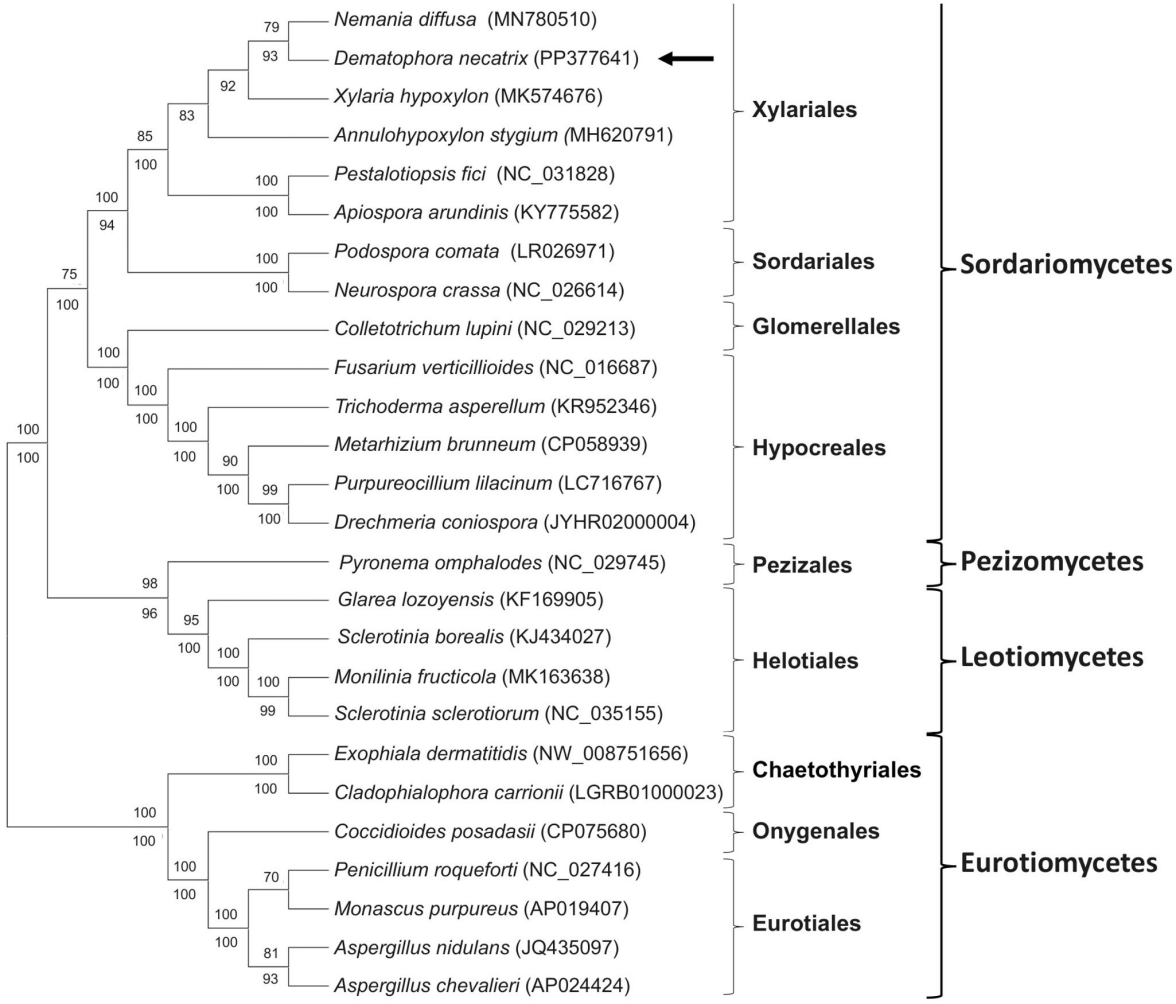
Maximum likelihood (ML) phylogenetic tree showing the relationships between *dematophora necatrix* strain CMW50482 and other members of the ascomycota, which were inferred from the concatenated amino acid sequences of the 14 conserved protein-coding genes encoded on the mitogenome. Similar clustering patterns were observed in our Neighbor-Joining (NJ) phylogeny. ML bootstrap support values are indicated above the nodes, while NJ bootstrap support values are indicated below the nodes. Accession numbers are indicated after the species names. Species used include the following: *Xylaria hypoxylon* (MK574676) (Zhou et al. 2019), *Dematophora necatrix* (PP377641) (this paper), *Nemania diffusa* (MN780510) (Tang et al. 2020), *Annulohypoxylon stygium* (MH620791) (Deng et al. 2018), *pestalotiopsis fici* (NC_031828) (unpublished), *Apiospora arundinis* (KY775582) (Yuan et al. 2019), *Podospora comata* (LR026971) (Unpublished), *Neurospora crassa* (NC_026614) (Monteiro et al. 2021), *Colletotrichum lupini* (NC_029213) (Pszczółkowska et al. 2020), *Fusarium verticillioides* (NC_016687) (Al-Reedy et al. 2012), *Trichoderma asperellum* (KR952346) (Unpublished), *Metarhizium brunneum* (CP058939) (Unpublished), *Purpureocillium lilacinum* (LC716767) (Unpublished), *Drechmeria coniospora* (JYHR02000004) (Unpublished), *Glarea lozoyensis* (KF169905) (Youssar et al. 2013), *Monilinia fructicola* (MK163638) (Unpublished), *Sclerotinia borealis* (KJ434027) (Mardanov et al. 2014), *Sclerotinia sclerotiorum* (NC_035155) (Unpublished), *Pyronema omphalodes* (NC_029745) (Unpublished) and *Aspergillus chevalieri* (AP024424) (Kadooka et al. 2021).

The ML an NJ phylogenies grouped *D. necatrix* with the Xylariales, where it was more closely related to members of the Xylariaceae (i.e. *Nemania diffusa* and *Xylaria hypoxylon*) than to taxa from other families (Figure 3). This close relationship was also evident from the syntenic nature of their mitogenomes (Figures S2B, S2C and SD). Like *D. necatrix*, the *N. diffusa*, *Annulohypoxylon stygium*, and *Apiospora arundinis* mitogenomes also contained *rps3* (albeit within the borders of *rnl*), while the *N. diffusa* and *Pestalotiopsis fici* mitogenomes also contained the *nat1* gene. Additionally, most of the *D. necatrix* mitogenome was non-coding and/or represented by introns, which is similar to other Xylariales. These similarities were despite gene losses in *X. hypoxylon* (Zhou et al. 2019) and *A. arundinis* (GenBank accession KY775582), and a large inversion in the *N. diffusa* mitogenome (Tang et al. 2020).

## Discussion and conclusion

The *D. necatrix* mitogenome closely resembles those published for other members of the Xylariales (Deng et al. 2018; Zhou et al. 2019; Tang et al. 2020). As in these fungi, the *D. necatrix* mitogenome encoded all of its protein-coding and rRNA genes in the same order and orientation. Likewise, the bulk of the *D. necatrix* tRNA genes occurred in clusters between the *rns* and *nad6* genes, and between the *nrl* and *nad2* genes.

Two notable protein-coding genes annotated in the *D. necatrix* mitogenome are *rps3* and *nat1*. In fungi, the *rps3* gene is often cycled between the nuclear and mitochondrial genomes by mobile genetic elements (Wai et al. 2019), and its product is a vital component of many cellular processes (Graifer et al. 2014; Medina et al. 2020). Not much is known about N-acetyltransferase-encoding genes such as *nat1*, but they have been implicated in mitochondrial turnover and the detoxification of plant defence compounds (Sharma et al. 2020). Therefore, these genes are potential targets for studies aiming to explore the molecular basis of pathogenesis in *D. necatrix*.

The large number of introns predicted in the *D. necatrix* mitogenome is consistent with previous reports from members of the Xylariales (Zhang et al. 2017; Deng et al. 2018) and Sordariomycetes (Medina et al. 2020). Indeed, these elements are implicated in the size variation and expansion of fungal mitogenomes (Wu et al. 2015). As expected for fungi (Mukhopadhyay and Hausner 2021), the *D. necatrix* mitogenome also contained more group I introns than group II introns. Due to their impact on the overall architecture of the mitogenome (Mukhopadhyay and Hausner 2021), intron activity may also impact the overall biology of the fungus harboring them. In certain fungi, for example, a particular allele of the group I type D intron occurring in *cob* has been shown to confer resistance to QoI (quinone outside inhibitor) fungicides (Cinget and Bélanger 2020).

The mitogenome assembled and characterized in this study provides many opportunities to improve our understanding of the biology and ecology of *D. necatrix* in South Africa. Apart from providing a sound foundation from which to explore the role of this organelle in the biology of the species, our findings would also serve as a valuable resource for exploring the genetic diversity and population biology of this important pathogen.

## Ethical approval

The Ethics Committee of the Faculty of Natural and Agricultural Sciences (NAS) at the University of Pretoria (Pretoria, South Africa) approved the work conducted in this study (reference number: NAS173/2020).

## Supplementary Material

Supplementary.docx

## Data Availability

The genome sequence data that support the findings of this study are openly available in GenBank of NCBI at https://www.ncbi.nlm.nih.gov/. The associated BioProject, BioSample, Genbank, and SRA numbers are PRJNA884201, SAMN31015769, PP377641 and SRR28283158.
