# Supplementary material for: Complete mitochondrial genome sequence of the white root rot pathogen Dematophora necatrix (Xylariaceae: Xylariales)
Source: Mitochondrial DNA B Resour. 2024 Sep 12;9(9):1207–12. doi: 10.1080/23802359.2024.2403411 (PMC11404375; doi:10.1080/23802359.2024.2403411)
Supplement: Supplementary.docx [file TMDN_A_2403411_SM0198.docx]

**Supplementary Materials**


**Figure S1.**

Read coverage depth map of *Dematophora necatrix* (Strain CMW50482) was created using GetOrganelle v3.0 (Jin et al. 2018).

**Figure S2.** Mitogenome comparison between *Dematophora necatrix* and other members of the Xylariales. **A:** Overview of the sizes and coding content of the respective genomes. Genes typically encoded on the mitogenome are *cox1*,*2*,3, *atp4*,*8*,*9*, *cob*, *nad1*,*2*,*3*,*4*,*4L*,*5*,*6*, *rnl* and *rns*, as well as several tRNA genes (indicated in parentheses). Other ORFs (open reading frames) include those encoding *rps3* and *nat1*, as well as predicted hypothetical proteins not associated with introns. Introns include those classified as group I and group II (^#^ indicates those in which introns were not classified to the two groups). **B:** Order of conserved genes and position of the two main clusters of tRNA genes (indicated with circled digits). The position of *rps3* within the *rnl* gene is shown with *, and in *D. necatrix* it is located between *rnl* and tRNA gene cluster 1 (not shown). The region from *nad3* to *cox1* in *Nemania diffusa* is encoded on the complementary strand. The order of the tRNA genes encoded in clusters 1 and 2 are shown in panel **C**, while those occurring at less conserved positions are shown in panel **D**. Each tRNA gene is indicated with the single letter code of its corresponding amino acid (i.e., alanine=A, arginine=R, asparagine=N, aspartic acid=D, asparagine or aspartic acid=B, cysteine=C, glutamic acid=E, glutamine=Q, glycine=G, histidine=H, isoleucine=I, leucine=L, lysine=K, methionine=M, phenylalanine=F, proline=P, serine=S, threonine=T, tryptophan=W, tyrosine=Y, valine=V).

**Table S1.** Position of group I and II introns in the *Dematophora necatrix* mitogenome (the position of genes encoding LAGLIDADG or GIY-YIG homing endonucleases are shown in blue).

| **Start** | **Stop** | **Feature** | **Intron type/Endonucleases type** |
| --- | --- | --- | --- |
| 2757 | 6578 | intron | Group IC2 |
| 2759 | 4036 | gene | LAGLIDADG |
| 17679 | 18828 | intron | Group IB |
| 17679 | 18563 | gene | GIY-YIG |
| 18862 | 20435 | intron | Group IC2 |
| 19472 | 20368 | gene | LAGLIDADG |
| 20493 | 24423 | intron | Group IA |
| 20896 | 22077 | gene | LAGLIDADG |
| 22441 | 23835 | gene | LAGLIDADG |
| 24658 | 26412 | intron | Group IC2 |
| 25337 | 26374 | gene | GIY-YIG |
| 26453 | 28134 | intron | Group IC2 |
| 26888 | 28117 | gene | GIY-YIG |
| 29105 | 30702 | intron | Group IC1 |
| 29732 | 30100 | gene | LAGLIDADG |
| 31057 | 32484 | intron | Group IC2 |
| 31057 | 32391 | gene | LAGLIDADG |
| 32575 | 38655 | intron | Group I |
| 33932 | 35215 | gene | GIY-YIG |
| 35743 | 38127 | gene | LAGLIDADG |
| 38803 | 40211 | intron | Group IB |
| 40347 | 42841 | intron | Group ID |
| 41739 | 42749 | gene | LAGLIDADG |
| 43046 | 46152 | intron | Group IB |
| 43049 | 44977 | gene | LAGLIDADG |
| 45126 | 46106 | gene | LAGLIDADG |
| 48410 | 50229 | intron | Group IB |
| 48773 | 50200 | gene | LAGLIDADG |
| 50422 | 51643 | intron | Group ID |
| 50422 | 51312 | gene | GIY-YIG |
| 55905 | 57272 | gene | Group IB |
| 56105 | 57031 | gene | LAGLIDADG |
| 81378 | 84830 | intron | Group IB |
| 81380 | 82396 | gene | LAGLIDADG |
| 85046 | 86296 | intron | Group IB |
| 85218 | 85649 | gene | GIY-YIG |
| 87408 | 89101 | intron | Group IC2 |
| 87410 | 88813 | gene | LAGLIDADG |
| 91389 | 92730 | intron | Group IB |
| 91390 | 92445 | gene | LAGLIDADG |
| 92908 | 94702 | intron | Group IC2 |
| 93363 | 94550 | gene | GIY-YIG |
| 94754 | 97890 | intron | Group IC2 |
| 95624 | 96331 | gene | GIY-YIG |
| 96473 | 97714 | gene | GIY-YIG |
| 102483 | 103294 | intron | Group II |
